# Molecular mechanism of Fast Endophilin-Mediated Endocytosis

**DOI:** 10.1042/BCJ20190342

**Published:** 2020-06-26

**Authors:** Alessandra Casamento, Emmanuel Boucrot

**Affiliations:** 1 Institute of Structural and Molecular Biology, University College London, Gower Street, London WC1E 6BT, U.K.; 2 Institute of Structural and Molecular Biology, Birkbeck College, Malet Street, London WC1E 7HX, U.K.

**Keywords:** Clahtrin-independent, endocytosis, endophilin, FEME, trafficking

## Abstract

Endocytosis mediates the cellular uptake of micronutrients and cell surface proteins. Clathrin-mediated endocytosis (CME) is the housekeeping pathway in resting cells but additional Clathrin-independent endocytic (CIE) routes, including Fast Endophilin-Mediated Endocytosis (FEME), internalize specific cargoes and support diverse cellular functions. FEME is part of the Dynamin-dependent subgroup of CIE pathways. Here, we review our current understanding of the molecular mechanism of FEME. Key steps are: (i) priming, (ii) cargo selection, (iii) membrane curvature and carrier formation, (iv) membrane scission and (v) cytosolic transport. All steps are controlled by regulatory mechanisms mediated by phosphoinositides and by kinases such as Src, LRRK2, Cdk5 and GSK3β. A key feature of FEME is that it is not constitutively active but triggered upon the stimulation of selected cell surface receptors by their ligands. In resting cells, there is a priming cycle that concentrates Endophilin into clusters on discrete locations of the plasma membrane. In the absence of receptor activation, the patches quickly abort and new cycles are initiated nearby, constantly priming the plasma membrane for FEME. Upon activation, receptors are swiftly sorted into pre-existing Endophilin clusters, which then bud to form FEME carriers within 10 s. We summarize the hallmarks of FEME and the techniques and assays required to identify it. Next, we review similarities and differences with other CIE pathways and proposed cargoes that may use FEME to enter cells. Finally, we submit pending questions and future milestones and discuss the exciting perspectives that targeting FEME may boost treatments against cancer and neurodegenerative diseases.

## Introduction

**Figure 1.**
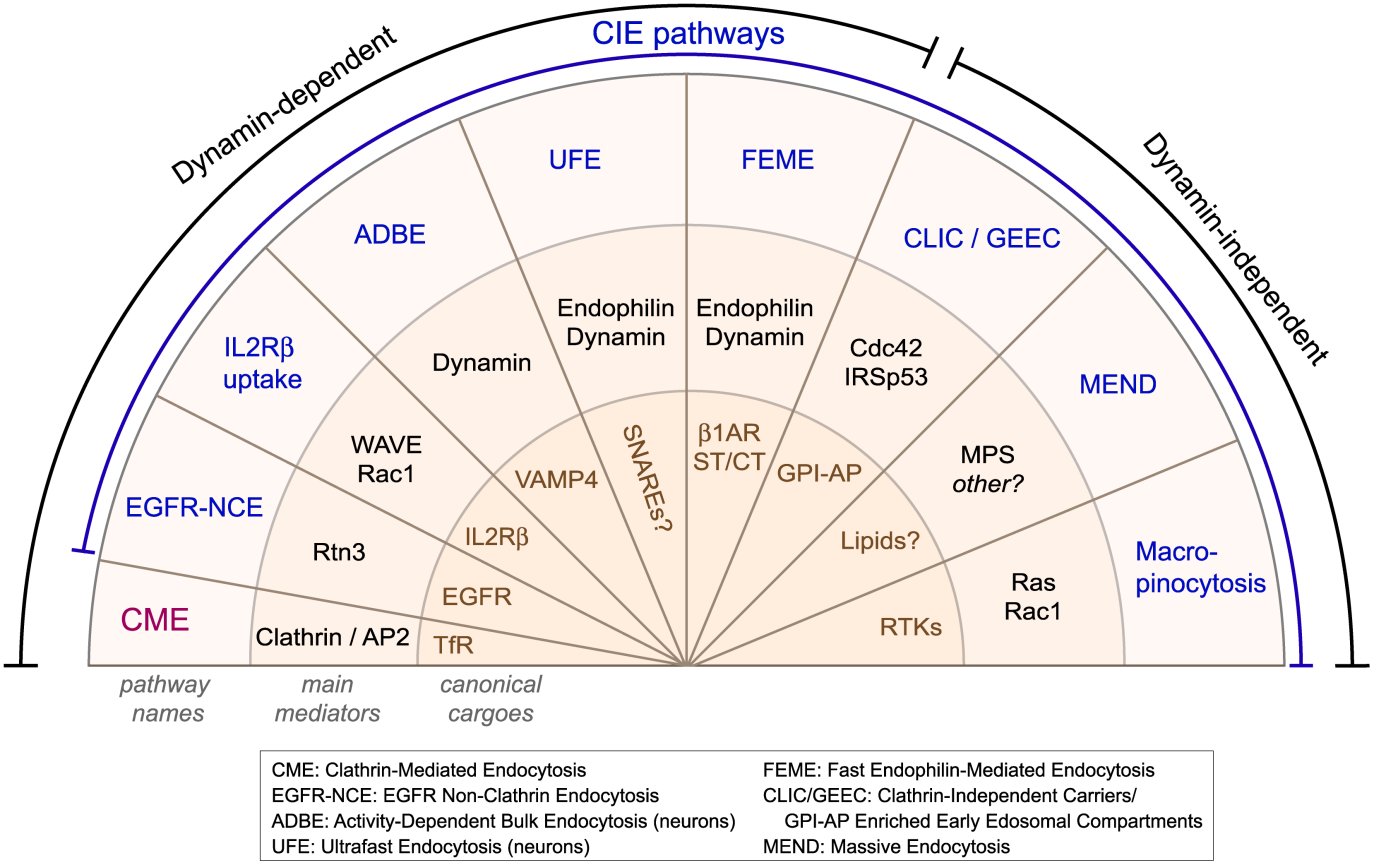
Clathrin-independent endocytic pathways. Clathrin-mediated endocytosis (CME) is the house-keeping pathway in resting cells. It is mediated by Clathrin and the tetrameric adaptor AP2 and its canonical cargo is Transferrin Receptor (TfR). Clathrin-independent endocytosis (CIE) is composed of Dynamin-dependent and Dynamin-independent pathways. EGFR Non Clathrin Pathway (EGFR-NCE) is regulated by Reticulon-3 (Rtn3) and internalizes Epidermal Growth Factor Receptor (EGFR) upon low doses of EGF. IL2Rβ uptake is a constitutive CIE pathway that internalizes IL2Rβ and γ under the control of Rac1 and WAVE. Activity-Dependent Bulk Endocytosis (ADBE) is controlled by Dynamin and internalizes VAMP4 and large patches of membranes upon high stimuli in neurons. Ultrafast Endocytosis (UFE) mediates the recycling of synaptic vesicle components (SNAREs?) in 50–100 ms following action potential in neurons. It is regulated by Endophilin, Dynamin and Synaptojanin. Fast Endophilin-Mediated Endocytosis (FEME) internalizes cargoes such as the β1-adrenergic receptor (β1AR) in 5–10 s following their stimulation, in an Endophilin- and Dynamin-dependent manner. Shiga toxin (ST) and cholera toxin (CT) can highjack FEME to enter cells, but can also use other CIE pathways. The Clathrin-Independent Carriers (CLIC)/GPI-anchored proteins (GPI-AP)-Enriched Early Endosomal Compartments (GEEC) pathway is a high capacity, Dynamin-independent, endocytic route, triggered by the extracellular clustering of GPI-AP, glycosylated proteins or lipids by Galectin-3. It is controlled by Cdc42, GRAF-1 and IRSp53. Massive Endocytosis (MEND) is the significant uptake of membrane induced upon Ca^2+^ and PI3 kinase signaling, mediated by membrane phase separation (MPS). Macropinocytosis is activated by strong and sustained Receptor Tyrosine Kinase (RTKs) signaling and form large (up to 20 µm) vacuoles upon the folding of membrane projections back to the cell surface.

Cell surface transmembrane proteins and extracellular material too large to diffuse through the membrane bilayer or be transported by channels and transporters are internalized through membrane-bound carriers, a process called endocytosis. The various endocytic pathways that exist form parallel portals of entry into cells. Clathrin-mediated endocytosis (hereafter, CME) is the best-characterized and the constitutively active and dominant uptake mechanism to support housekeeping functions in all eukaryotic cells [1–3]. There is evidence of several distinct mechanisms of Clathrin-independent endocytosis (CIE), including the CLIC/GEEC pathway (Clathrin-independent carriers (CLIC), glycosylphosphatidylinositol-anchored proteins (GPI-AP)-enriched early endosomal compartments (GEEC)), IL2Rβ uptake, EGFR non-clathrin endocytosis (EGFR-NCE), Fast Endophilin-Mediated Endocytosis (FEME), massive endocytosis (MEND), macropinocytosis, as well as neuron-specific activity-dependent bulk endocytosis (ADBE) and ultrafast endocytosis (UFE) [4–9] (Figure 1). Caveolae can in theory contribute to Clathrin-independent uptake. Even though caveolae can bud from the cell surface, few if any cargoes rely on them for their uptake [10,11]. The pathways are defined by characteristic endocytic carrier morphologies, by cytosolic markers, by ligands and receptors (collectively called ‘cargoes’) that use them to enter cells or by the speed of completion [6–9,12]. Overall, CIE pathways were observed in a wide variety of *in vitro* cell lines, *ex vivo* primary cells, as well as *in vivo* in mouse, fly, worm, plant and yeast. Several CIE processes are not constitutively active and perform specific or temporally regulated cellular functions. These range from bulk lipid and extracellular protein uptake and removal of activated receptors from the cell surface, to the control of cell polarization, spreading and migration [4–9].

Some CIE pathways such as UFE, FEME and MEND are quicker than CME and function in physiological processes requiring fast (<10 s) internalization from the plasma membrane (Figure 1), such as reaction to stress hormones (‘fight-or-flight’ response) and receptor hyper-stimulation, chemotaxis or compensatory endocytosis following exocytosis of synaptic or hormone-containing vesicles [6,13]. Many pathogens hijack CIE pathways to infect cells: these include over twenty viruses (including Ebola, HIV, Lassa, Herpes, Dengue and SV40 viruses), some bacteria, prions and bacterial toxins (including cholera and Shiga toxins, Streptolysin O and VacA [14,15]. Finally, deregulations of CIE have been reported during cancer, lysosomal storage disease or atherosclerosis.

FEME was recently added to the CIE family of pathways (Figure 1) and some of its molecular steps are now established [16–18]. The existence of specific cargoes, endocytic carrier attributes and cytoplasmic markers enabled the swift elucidation of key molecular and regulatory mechanisms. In addition, although FEME was only defined few years ago, some of its cargoes have been studied for longer, in particular cholera and Shiga toxins that can hijack FEME to infect cells [17]. These were instrumental in establishing the scission mechanism of FEME [17,19]. In this review, we outlay our current understanding of FEME and compare and contrast it with other CIE pathways. We also discuss potential cargoes that may use FEME and propose future milestones in the understanding of the pathway.

## Hallmarks of FEME

FEME is a Dynamin-dependent, Clathrin-independent endocytic route marked and regulated by Endophilin. Part of the BAR domain superfamily, Endophilin consists of five proteins in human: Endophilin A1, A2, A3, B1 and B2. Only A1, A2 and A3 function in FEME (and thus collectively referred to as ‘Endophilin’), while Endophilin B1 and B2 are involved in autophagy and mitochondrial dynamics [20]. Endophilin A2 is ubiquitously expressed whereas Endophilin A1 and A3 are tissue restricted (mostly brain, kidney and testes). However, compensations exist upon genetic ablation of any one of them [21] and the depletion of all three forms is required to block FEME [16]. Endophilin functions in CME by supporting Dynamin-induced scission and Synaptojanin-mediated Clathrin uncoating [21]. However, Endophilin is redundant in CME (its roles can be sustained by Amphiphysin and SNX9 [22,23]), but essential in FEME. Moreover, Endophilin is only detected on a fraction of forming Clathrin-coated vesicles [24], confirming a likely peripheral role in CME.

Endophilin features two functional domains: a BAR domain — banana-shaped concave structure mediating protein dimerization and membrane curvature — and a SRC Homology 3 (SH3) domain that binds to proline-rich regions in target proteins [20,25,26]. The BAR domain contains two amphipathic helices H0 and H1 (H0 is at the N-terminus, making a ‘N-BAR domain’ [25]), which promote membrane curvature sensing, stabilization and formation beyond that of a regular BAR domain. Endophilin is the main marker and regulator of FEME. It performs three functions: (i) membrane curvature promoted by the N-BAR domain; (ii) cargo engagement through its SH3 domain; and (iii) membrane scission, upon recruitment of Dynamin and actin via interactions mediated by the SH3 domain and membrane friction and lipid diffusion barrier by its N-BAR domain.

FEME is defined by the formation of Clathrin-negative, Endophilin-positive endocytic carriers that bud from the plasma membrane upon stimuli. Hallmarks of FEME to date are:

(1) it is not constitutively active, but is rapidly (few seconds) triggered by the activation of receptors by their cognate ligands [16];
(2) it requires the pre-enrichment of Endophilin into discrete clusters on the plasma membrane, prior to receptor activation [18];
(3) it is prominent at the leading edge of cells but also occurs on basal and dorsal cell surfaces [16];
(4) FEME carriers are small (∼1 µm), pleiotropic, tubulo-vesicular Endophilin positive assemblies (EPAs) found in the cytosol [16]. Unlike CME, where the Clathrin coat is lost soon after budding, Endophilin remains on FEME carriers until fusion with early endosomes;
(5) to date, there are 16 confirmed FEME cargoes: β1 and α2a adrenergic receptors, Dopamine receptors 3 and 4, Muscarinic Acetylcholine receptor 4, EGFR, HGFR, VEGFR, PDGFR, NGFR, IGFR, tetrameric IL2R, PlexinA1 and ROBO1, as well as cholera and Shiga toxins [16,17,27,28]. However, many of these cargoes can also use other endocytic pathways to enter cells;
(6) membrane scission requires the synergy between Endophilin, Dynamin and actin [17,19];
(7) cytosolic FEME carriers move retrogradely (towards the perinuclear area) on microtubules, powered by Dynein [17,28];
(8) it is negatively regulated by Cdk5 and GSK3β [28]. Acute inhibition of the kinases is sufficient to induce FEME.

Several of these characteristics must be met to establish that FEME is the portal of entry of candidate cargoes. Technical and protocol details are important to preserve and detect FEME carriers.

## Mechanism of FEME carrier formation

### Priming

The promptness of the pathway to activate upon receptor stimulation is explained by the priming cycle of FEME. In resting cells, Endophilin is pre-enriched into small patches at discrete locations on the plasma membrane. The precession of Endophilin on the membrane before cargo sorting is critical for prompt carrier formation. In resting cells, the vast majority of Endophilin molecules are auto-inhibited in the cytosol, through intra-dimer, inter-monomer interactions between H0 helices and SH3 domains from different subunits within homodimers [29]. As engagement of the SH3 domain with a PRM is necessary, and sufficient, to relieve the auto-inhibition, Endophilin must be targeted to the membrane by other proteins *in vivo* [18,22]. Only then can its N-BAR domain can bind to the membrane to sense, stabilize or induce local membrane curvature.

**Figure 2.**
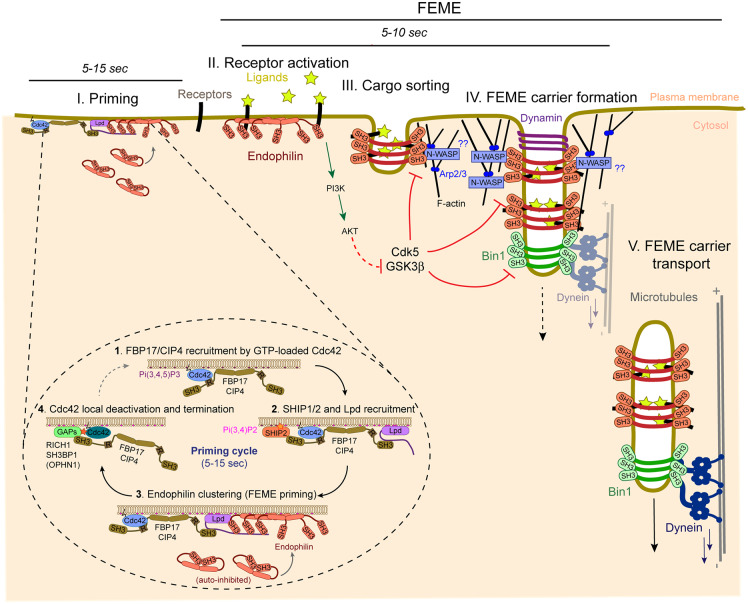
Molecular steps of FEME carrier formation. Endophilin pre-enrichment on the plasma membrane (Stage I) is a prerequisite for FEME and mediates the promptness of the pathway to activate upon stimulation. FEME in itself only starts upon receptor activation and thus, corresponds to Stages II to V. Stage I. Plasma membrane priming cycle. Step 1, at high Pi(3,4,5)P_3_ patches, active GTP-loaded Cdc42, recruits FBP17 and CIP4 through their REM domains (R). Step 2, FBP17 and CIP4 cluster 5′-phosphatases SHIP1 and 2 as well as Lamellipodin (Lpd) through their SH3 domains. Lpd is further stabilized by Pi(3,4)P_2_ locally produced by SHIP1/2, Step 3, Endophilin is recruited from the cytosol (where it is autoinhibited) and concentrated by Lpd. From there, pre-enriched Endophilin mediates prompt FEME carrier formation upon cargo activation. In absence of cargo activation, the FEME priming complex aborts and disassembles (Step 4), upon local Cdc42 deactivation by the GAPs RICH1, SH3BP1 and Oligophrenin (OPHN1). Stage II. FEME activation upon cargo stimulation. Activation of receptors by their ligands stabilizes pre-enriched Endophilin patches and starts FEME. The exact signals driving this stage are still unclear. However, stages II to IV are inhibited by Cdk5 and GSK3β, which hold off FEME. Upon receptor activation, PI3K, AKT and ERK signaling inactivate GSK3β, thereby releasing its inhibitory action and activating FEME. Stage III. Cargo sorting. Activated receptors are recruited to nascent FEME pits either through direct binding to Endophilin (e.g. β 1 adrenergic receptor) or through binding of adaptor proteins (e.g. Cbl–CIN85 complex recruiting EGFR). Stage IV. FEME carrier formation. Local addition of Endophilin molecules by activated cargoes trigger membrane curvature, likely supported by local actin polymerization. Dynamin is recruited by Endophilin but excluded from the main body of the tubules, likely to avoid premature membrane scission. Detachment of FEME carrier is achieved by the synergy of GTP-dependent membrane pinching by Dynamin, membrane tension imposed by local actin cytoskeleton and friction-driven scission (FDS) lipid diffusion barrier by the N-BAR domains of Endophilin. Stage V. FEME carrier transport. Swiftly after budding, FEME carriers are rapidly transported on microtubules by Dynein, recruited onto the tubules by Bin1. The precise timing of Dynein loading on FEME carriers (Stage IV or V) is not yet clear.

Endophilin clusters on the plasma membrane of resting cells are dynamic and last from 5 to 15 s before abortion (dissolution without endocytosis). As they rely on Pi(3,4)P_2_ production from Pi(3,4,5)P_3_, they are prominent at the leading edge of migrating cells, but are also numerous on the ventral and dorsal surfaces of confluent monolayers [16,18]. The priming cycle is initiated by membrane-bound, active, GTP-loaded Cdc42, which recruits FBP17 and CIP4, via their REM (also called HR1) motifs (Figure 2, Stage I). FBP17 and CIP4 concentrate on the membrane upon binding of their F-BAR domains and heterodimerization [18]. The tissue-restricted TOCA1 might be involved in this step in some cells as it heterodimerizes with both FBP17 and CIP4, and all three are functionally redundant in FEME [18]. Next, the SH3 domains of FBP17 and CIP4 recruit both SHIP1/2 phosphatases and Lamellipodin. SHIP1 and 2 are 5′-phosphatases that locally hydrolyze Pi(3,4,5)P_3_ into Pi(3,4)P_2_, which is then bound by the PH domain of Lamellipodin, further stabilizing the latter (Figure 2, Stage I). Lamellipodin has multiple PRMs to which Endophilin bind, and thus it concentrates many copies into patches [30]. These events are obligatory steps which must happen in sequential manner. Depletion of any of these proteins stalls the process downstream of the step targeted, which invariably block FEME carrier formation upon receptor activation [18].

Once Endophilin is pre-enriched into clusters, receptor activation, or absence thereof, results in two opposite fates:

- Upon ligand-receptor interaction and downstream signaling, Endophilin initiates cargo capture and local membrane curvature (Figure 2, Stage II to V), subsequent mechanisms are described below.
- In the absence of receptor stimulation, local Cdc42 deactivation by GTPase-activating proteins (GAPs) RICH1, SH3BP1 and Oligophrenin induce the disassembly of the priming complex and abortion of the Endophilin patches [18] (Figure 2, Stage I). New clusters of Endophilin reform stochastically nearby, repeatedly priming the membrane for FEME.

### Cargo selection

So far, FEME cargoes are a subset of the amine GPCRs (β1- and α2a-adrenergic receptors, Dopamine receptors 3 and 4 and Muscarinic Acetylcholine receptor 4), RTKs (EGF, HGF, VEGF, PDGF, NGF and IGF-1 receptors), cytokine receptors (tetrameric Interleukin receptor 2, IL-2R) and axon guidance receptors (Plexin A1 and ROBO1) [16,27,28]. In addition, the pathways is highjacked by cholera and Shiga toxins [17]. Many of these cargoes and toxins can enter cells through other endocytic pathways (CME, CLIC/GEEC, EGFR-NCE or macropinocytosis) [31–33]. To date, only the β1-adrenergic receptor (β1-AR) appears to rely exclusively on FEME for cellular entry [16,18,34]. However, inhibition of FEME induces the accumulation of many of these receptors at specific cellular locations (e.g. leading edge), even though their global uptake may not be affected [16].

In all instances studied so far, cargoes are sorted into FEME carriers upon direct or indirect binding to Endophilin (Figure 2, Stage II). The Endophilin SH3 domain binds directly to proline-rich regions present in intercellular loops of the identified GPCRs [16,35]. This contrasts with growth factor and axon guidance receptors, which are sorted through the binding of cytosolic adaptors. Both EGFR and HGFR are sorted upon binding of CIN85 to Endophilin (which are bridged to the receptors by Cbl) [36,37]. Recently, Plexin A1 and ROBO1 were found to bind to Endophilin through Collapsin Response Mediator Protein 4 (CRMP4) and srGAP1, respectively [27,28].

### Membrane curvature and carrier formation

How membrane curvature is generated is still unclear. Endophilin and its N-BAR domain are required, as in their absence no FEME carriers are produced [16,18]. However, the transition between the priming patches of Endophilin (which do not appear to generate meaningful curvature) and the shaping of tubules and vesicles following receptor activation is not understood. Either the priming clusters do not reach a critical local concentration of Endophilin required to deform the plasma membrane — and receptor activation boosts the number of Endophilin molecules over the threshold. Or, a so far unidentified mechanism is controlling membrane curvature by the N-BAR domain until activation. The recent finding that Bin1, another N-BAR protein is present on virtually all FEME carriers [28], opens the possibility of a third alternative, whereby the number of Endophilin and Bin1 molecules matter. However, Bin1 is also present on most priming patches [28], thus its recruitment is not the trigger in itself. Actin polymerization, which is required for carrier production [16], may have a role in inducing or stabilizing membrane curvature during tubule formation (Figure 2, Stage III). But isolating its role during priming (potential corralling of the patches), membrane curvature formation, membrane scission and potential short distance cytosolic transport is technically challenging.

Shiga and cholera toxins, which induce inward membrane bending from outside of the cells [38], revealed that if the initial curvature is provided, it is sufficient for Endophilin to take over and mediate the next steps (membrane scission and carrier transport) [17]. Thus, perhaps the number of Endophilin molecules recruited on toxin-induced tubules is lower than that on canonical FEME carriers, and the threshold hypothesis is the main driver.

### Membrane scission

Detachment of the carriers requires the co-ordinated action of Dynamin, actin and N-BAR domain of Endophilin (and perhaps Bin1) [17] (Figure 2, Stage IV). Dynamin is a GTPase that mediates membrane scission in a large number of endocytic processes, including CME [39]. It is recruited to the neck of endocytic carriers by the SH3 domains of several BAR domain proteins, including Endophilin and Bin1 [22,40]. There, the co-ordinated hydrolysis of GTP induces conformation changes that drive membrane fission [41]. Because Endophilin can also inhibit Dynamin activity [42], local stoichiometry is likely key in determining the location of the scission event (Figure 2, Stage IV). By locally blocking Dynamin, Endophilin might avoid premature cuts and allow the forming tubules to grow and package enough cargoes before detachment. It is now clear that Dynamin is necessary but not sufficient, and a synergy with local membrane tension provided by local actin scaffold and friction-driven scission (FDS) by N-BAR domains is required [17,19]. The FDS mechanism arises from the frictional barrier for lipid diffusion created by oligomerized BAR domains [19] (Figure 2, Stage IV). One remaining question is whether forces induced by Dynein are required for membrane scission. *In vitro*, typical scission assays use molecular motors (in that case Kinesins) to pull membrane tubes away from giant unilamellar vesicles (GUVs), thereby mimicking the neck of a forming endocytic carrier. However, when exactly is Dynein recruited onto FEME carriers and how much is it required for membrane scission *in vivo* is not clear yet.

### Cytosolic transport

Actin polymerization is required for FEME carrier formation and perhaps for the first few microns displacement into the cytosol. Endophilin binds to N-WASP and to the WAVE complex [18,43], but more work is needed to understand the molecular events recruiting actin to budding FEME carriers. As for other CIE carriers, Dynein powers long-range and fast movements of FEME carriers on microtubules [17,28,44] (Figure 2, Stage V). Bin1 recruits Dynein to the carriers [28] but molecular details are lacking to date.

### Regulatory mechanisms

Because FEME is not constitutively active, many regulatory mechanisms must exist to hold it off and activate it on demand. This is also apparent in the diversity in FEME activity displayed by different cell types. While ligand binding triggers internalization of cargo receptors, some cell types present robust spontaneous FEME, which is apparent in resting cells that were not activated by any ectopic ligand [28]. This is due to high levels of growth factors in their regular culture media. Because such spontaneous FEME is lowered by serum starvation (removal of growth factors) and, conversely, activated upon stimulation with extra serum [16,28], it suggests a link between some cell signaling events and FEME activity.

The levels of Pi(3,4)P_2_, and that of the sequential precursors Pi(4,5)P_2_ and Pi(3,4,5)P_3_, are tightly controlled [45,46]. As such, phosphoinositides kinases and phosphatases that balance their production are regulating FEME. By producing the local Pi(3,4)P_2_ required for the priming mechanism, SHIP1 and 2 phosphatases have a direct activating effect on the pathway. Upstream, class I PI3 kinases are also positive regulators because they boost the levels of Pi(3,4,5)P_3_, which is required for Pi(3,5)P_2_ production by SHIP1/2 [46,47]. At the reverse, PTEN and Synaptojanin are negative regulators because they reduce local levels of Pi(3,4,5)P_3_ and Pi(4,5)P_2_, respectively [46,48]. Beyond phosphoinositides, FEME is also controlled by several phosphorylations imposed on cargoes, Dynamin and Dynein by Cdk5 and GSK3β [28]. Cdk5 and GSK3β are well known to play important roles in regulating endocytosis. They do so by phosphorylating Amphiphysin, Dynamin and other endocytic proteins in synapses [49]. Dynamin-1 is phosphorylated at Ser778 by Cdk5, followed by that of Ser774 by GSK3β [50,51]. The phosphorylation of Ser778 blocks the recruitment of Dynamin-1 by binding partners such as Endophilin, and that of Ser774 inhibits Dynamin-1 activity. Interestingly, these phosphorylations dampen dysregulated CME but activate ADBE [51,52], suggesting they might regulate a balance between the pathways. However, it is not known whether Dynamin-1 directly controls a cross-talk between FEME and CME. Both pathways also use Dynamin-2, which does not have a precise conservation of the Cdk5 and GSK3β phosphorylation sites and does not appear to be under the same control as Dynamin-1 [52]. But if acute activation of Dynamin-1 upon the inhibition of GSK3β triggers rapid, dysregulated CME [52], it appears to simply enhance FEME in its regular form [28]. Because Endophilin is phosphorylated by Src, ROCK and LRRK2 and FEME is blocked upon PAK1 and 2 inhibition [16,53–55], additional regulatory mechanisms remain to be understood. Of particular interest are the phosphorylations of Endophilin in its amphipatic Helix 0 (Thr14) by ROCK and Helix 1 (Thr73 and Ser75) by LRRK2, which hamper its membrane-binding ability [54–57]. However, to what extent this mechanism controls FEME remains to be investigated.

## Similarities and differences with other CIE pathways

Characteristics and mechanisms of endocytic carrier formation by CIE pathways have been reviewed elsewhere [7–9,12]. We focus here on highlighting analogies and disparities between FEME and other know CIE routes.

### Dynamin dependency

Because efficient mutants and excellent small compound inhibitors that block Dynamin exist, the requirement for an endocytic pathway is easy to establish and makes for a clear characteristic. The reliance on Dynamin splits the CIE pathways into two groups (Figure 1). FEME shares this dependency with the IL2Rβ uptake and EGFR-NCE, as well as ADBE and UFE in neurons. Even though FEME shares some similarities with UFE [58], it is clearly different phenomelogically or mechanistically to the other pathways [6,7] and thus, Clathrin-independence but Dynamin-dependency is not sufficient to identify FEME.

### Constitutive or triggered

A cardinal feature of FEME is its non-constitutive activity. It needs a signal to be triggered: receptors to be activated by their cognate ligands [16] (Figure 2, Stage II). This is shared with many other CIE pathways, which also are not constitutively active. CLIC/GEEC appears constitutive because it is detected in resting cells without any external stimuli [59]. However, it is inhibited by increased membrane tension and enhanced by the extracellular clustering of GPI-anchored proteins, or glycoproteins or glycolipids by galectins, toxins and viruses [60]. Although at different signal strengths, macropinocytosis and EGFR-NCE are activated upon growth factor stimulation [33,61], and ADBE and UFE are triggered upon axon depolarization [62,63]. Only the uptake of IL2Rβ and γ chains, and that of inactive GPCRs occur through constitutive Clathrin-independent routes [64,65].

### Role of actin

Actin polymerization is required for all CIE pathways but MEND (which appears to prevent it) [13,66]. However, molecular events controlling actin dynamics vary widely between different pathways. The main discriminators are the small G proteins RhoA, Rac1 and Cdc42, as they control different types of actin polymerization (branched or bundled). For example, the CLIC/GEEC pathway is Cdc42-dependent, whereas IL2Rβ uptake relies on RhoA and Rac1 (reviewed in [66]). CLIC maturation involves Cdc42- and N-WASP-mediated local actin polymerization, with Cdc42 being spatially and temporally controlled by ARHGAP10 [67]. Local generation of F-actin around clustered IL2Rβ or γ chains is promoted through the direct binding of the WAVE complex to the receptors, via WIRS motifs present in their cytoplasmic tails [68]. The receptors also induce PI3K-mediated enrichment of Pi(3,4,5)P_3_, followed by the sequential recruitment of RhoA, Rac1, Pak1, WAVE and N-WASP to mediate local cytoskeleton remodeling and membrane protrusions [69,70]. FEME is atypical in that it needs all three small G proteins, although probably at different time. It is clear that Cdc42 is required for priming, but nothing is known about RhoA and Rac1, other than blocking their activity inhibits FEME [16].

### Cargo specificity

Cargoes that enter cells independently of CME have been long used to identify and label CIE pathways [8,9,71]. As such, CLIC/GEEC, IL2Rβ uptake and EGFR-NCE are cargo specific, and there is a good understanding of the molecular steps leading to cargo selection [7] (Figure 1). Some other CIE processes, such as macropinocytosis, MEND, ADBE or UFE do not have known cargo-sorting mechanisms and internalize any proteins located on the patches of membrane being engulfed [7] (Figure 1). All internalized transmembrane proteins are then sorted in endosomes: recycled back to the cell surface or degraded in lysosomes. FEME is part of the first group (cargo-specific) because of the mechanisms explained above. However, it appears to have the particularity — only shared by IL2Rβ uptake — of internalizing only one type of cargo per carrier [16]. But studies carefully measuring cargo stoichiometry upon co-stimulation by several ligands are lacking to definitely conclude on that property.

### Shapes and speed of carrier formation

Morphologically, FEME carriers most resemble CLICs, which are tubules that often adopt a ‘C’ shape in the cytoplasm [72]. However, FEME tubules are more pleiotropic, as their length can vary from 100 nm to several microns [16]. UFE and IL2Rβ uptake produce rounded, uncoated vesicles of 60 nm and 500 nm in diameter, respectively [62,68], whereas ADBE, MEND and macropinocytosis generate larger vacuoles (from 500 nm to 10–20 µm in diameter). The speed of carrier formation places FEME between UFE, which form vesicles within 50 to 100 ms from stimuli, and other, slower, CIE processes.

## How to identify FEME

Establishing that a receptor or a ligand enters through FEME requires more than just inhibiting Endophilin. This is because of its additional role in CME [21], as well in potentially unidentified and unrelated processes. High confidence identification requires the combination of: (i) providing direct evidence that the candidate cargo is internalized into FEME carriers, (ii) showing that such uptake is triggered by ligand addition and (iii) that inhibition of Endophilin blocks cargo uptake [16].

Importantly, details of the protocol used are critical. Historically, uptake assays have been performed following a 30 min to 1 h pre-incubation at 4°C, prior to switching to 37°C for various amounts of time. This is to pre-bind ligands to their receptors exposed at the cell surface and to synchronize endocytosis. Other protocols call for uptake at room temperature instead of 37°C. However, these practices reduce membrane fluidity and may induce a phase transition, which will likely not reverse within the time frame of fast endocytic processes. Consistently, both UFE in synapses (which is Endophilin-dependent [58]) and FEME are only observed at physiological temperature (37°C for mammalian cells) [16,62]. An artificial increase in membrane fluidity by increasing the proportion of polyunsaturated lipids boosts Endophilin-mediated uptake [73].

Another step used to boost endocytosis and facilitate measurements is serum starvation for several hours (up to overnight) prior to endocytic assays. This was historically done to artificially increase the levels of receptors at the cell surface for early electron microscopy studies [74]. This practice has been routinely used in many experiments since. However, FEME is inhibited upon serum starvation, even as short as 30 min [16]. This is because growth factor removal increases GSK3β activity by depressing AKT and ERK-mediated inhibitory phosphorylation on Ser 9 [75]. Together with Cdk5, GSK3β negatively regulates FEME and thus its activation by serum starvation has lasting depression effects on FEME [28]. In addition, the artificial accumulation of receptors at the cell surface caused by serum starvation can change their mode of internalization and might favour their uptake by pathways that are not normally used [76]. Thus, to identify and measure FEME, it is critical to work in physiological conditions, without any serum starvation or temperature shifts.

To compensate for low and unsynchronized ligand binding to their receptors, direct incubation times at 37°C must be longer than the actual endocytic events. We found that 4 to 5 min incubation times to be sufficient for trigger FEME by all ligands tested to date. This does not mean that individual endocytic events take that long, as most of the time is used by the ligands to diffuse in the medium, to bind to their receptors and to activate them. Indeed, direct observation showed that individual FEME carriers take 5 to 15 s to form [16]. In line with the above considerations, warm fixation (e.g. pre-warmed PFA incubated at 37°C for the first 10 min) best preserves FEME carriers.

A word of caution should be said about ectopic expression of Endophilin and cargoes. Elevated levels will change the stoichiometry between FEME components and may induce artifactual events. Endophilin is auto-inhibited in the cytosol and opens up upon the binding of its SH3 domain to PRMs (of cargoes, Dynamin etc.) [18,22]. Higher levels of expression will induce an excessive membrane tubulation by the BAR domain and destabilize the balance required for FEME carrier formation. Likewise, overexpressing a receptor bears the risk of vastly outnumbering endogenous copies (most receptors are expressed at very low copy numbers [77]) and ‘leaking’ into non-natural endocytic routes. Endogenous tagging with fluorescent proteins using gene editing is an option and was performed successfully for Endophilin in Hep2β cells [23], save for the risk of dim signals and potential steric hindrance of the tag. Until now, our technique of choice has been the immunostaining of endogenous Endophilin and cargoes. Ideally, recombinant ligands or antibodies recognizing the ectodomains of endogenous cargo receptors (exposed to the media) should be added to the cells and their enrichment into FEME carriers determined by co-immunostaining of Endophilin post fixation [16]. Cargoes internalized into FEME carriers can be detected by confocal microscopy or electron microscopy.

To date, only genetic means can be used to block FEME with some specificity. Genetic inactivation of Endophilin using triple knock-down (TKD) or knock-out, or overexpression of the inactive mutant ΔH0-BAR domain works well [16], with the caveat mentioned above that CME (and perhaps other cellular processes) might be affected. The ΔH0-BAR mutant is the strict BAR domain (of either Endophilin A1, 2 or 3), lacking the first amphipathic helix, middle region and SH3 domain. It binds very poorly to membranes and should not bind to any interacting partners, but still heterodimerises with endogenous Endophilin [16]. Thus, it imposes a dominant-negative effect upon overexpression as it titrates out endogenous Endophilin and sequesters it in the cytosol. Inhibition of the priming mechanism that pre-enriches Endophilin at the plasma membrane before receptor activation is another strategy. This is achieved by FBP17, CIP4 and TOCA-1 triple knock-down, SHIP1 and 2 double knock-down or Lamellipodin depletion [18]. Overexpression of the Cdc42 GAPs that terminate the priming cycle (RICH1, SH3BP1 or OPHN1) are also effective at depressing FEME [18].

Although many small compound inhibitors block FEME, none, so far, are specific to the pathway. Dynamin inhibitors (e.g. Dynasore, Dyngo 4a, Dynole 34.2, or Chlorpromazine [78–81] are very effective at stopping FEME, but also block CME and other endocytic pathways. PI3K inhibitors work by inhibiting the production of Pi(3,4,5)P_3_, and thus Pi(3,4)P_2_, thereby abrogating the FEME priming mechanism. But PI3Ks work in many other cellular processes, including actin polymerization and parallel endocytic routes. Consistently, both actin poisons (Cytochalasin D, Latrunculin B, Jasplakinolide) and inhibitors of actin regulators (RhoA, Rac, Arp2/3 inhibitors) block FEME as well as other pathways [66]. Because a dynamic cycle of activation-inhibition of Cdc42 drives the priming of FEME, Cdc42 inhibitors can either be stimulatory or inhibitory (depending on concentrations and incubation times) [16,18], and thus are not a reliable way of controlling the pathway. Amongst chemicals historically used to inhibit CME, macropinocytosis and other CIE pathways, it is important to realise that many block FEME as well [16]. Thus, the phenotypes observed could be caused by the defect in FEME, instead, or in addition to, the other endocytic pathways studied.

Amiloride, and its more soluble analog 5-(*N*-ethyl-*N*-isopropyl) amiloride (EIPA), are the gold standard for inhibiting macropinocytosis, but they also block FEME [16]. Because they are inhibitors of the Na^+^/H^+^ exchanger, they depolarize the plasma membrane, thereby destabilizing several small GTPases, including Rac and Cdc42 [82]. PAK1 and 2 inhibitors also perturb macropinocytosis, in addition to IL2Rβ uptake and FEME [16,69]. High sucrose (hypertonic medium), potassium depletion, MDC, CPZ or PAO stop FEME in addition to CME [16]. Likewise, drugs depleting or sequestering cholesterol (MβCD, filipin, nystatin or simvastatin) have been used in thousands of publications as sole evidence that cargoes (receptors, toxins, viruses or bacteria) enter cells through ‘raft endocytosis’. However, because these drugs have pleiotropic effects — including abrogating FEME - some of these papers might need to be revised upon more rigorous investigation. Overall, small compound inhibitors should be used with caution in the study of FEME and cannot be the sole basis for conclusion. They must be complemented by other evidence, as explained above.

Means to boost FEME include hyperactivating the priming cycle by (moderately) overexpressing CIP4 or FBP17, depleting RHICH1+SH3BP1 or inhibiting PTEN (RNAi or small compounds) [16,18]. It can also be achieved by relieving the negative regulation by Cdk5 and GSK3β (RNAi or small compounds) [28]. Overexpression of Endophilin itself does not stimulate well FEME, as explained above.

Finally, the choice of cells is important. Some cell lines such as HeLa or Hek293 display little FEME compared with other cell types such as normal human retinal pigment epithelial cells (RPE1), primary skin fibroblasts or human umbilical vein endothelial cells (HUVEC) [28]. The differences in activity in different cell types can be 5 to 8 folds [28] and thus influence the ease of observing FEME.

## Cargoes that may use FEME

Equipped with recent insight, we can review past literature on cargoes that are known to enter cells in an Endophilin-dependent manner and discuss which ones could be using FEME. Several papers identified the binding of Endophilin to cargoes and concluded that the latters must enter cells through CME, but we now know that this is not necessarily the case. Of particular interest are cargoes that interact with Endophilin in a ligand-dependent manner, as it is a cardinal feature of FEME. For example, both CD36 and scavenger receptor A (SR-A) were immuno-precipitated by Endophilin only upon oxidized low-density lipoprotein (oxLDL) treatment [83]. Depletion of Endophilin increased the cell surface levels of CD36 and SR-A and decreased oxLDL uptake in macrophages [83] (Table 1). CME was proposed to be the pathway on the basis of two inhibitors, but more detailed characterization is required to rule out FEME. Other stimuli-induced binding to cargoes may include the interaction of Endophilin A1 and A2 with voltage-gated Ca^2+^ channels (L, N and P/Q types) [84] (Table 1). They were reported to do so at resting Ca^2+^ concentration (100–300 nM) through their linker regions between the BAR and the SH3 domain [84]. Endophilin was also reported to interact with the Chloride ClC-3 channel [85], the small-conductance calcium activated K_ca_2.3 channel [86] and the gamma-aminobutiric acid GABA receptor B [87], which is a ligand-gated Chloride channel (Table 1), but these interactions have not been followed up to date. The binding of Endophilin to several ion channels is intriguing because it is not believed that channels are actively endocytosed, and even less so rapidly in a ligand- or stimuli-dependent manner.

**Table 1:** Cargoes that may use FEME to enter cells

Putative FEME cargoes	Binds to Endophilin?	Endophilin-dependent uptake?	Ligand-dependent uptake?	References
CD36	Yes	Yes	Yes (oxLDL)	[83]
Scavenger Receptor A	Yes	Yes	Yes (oxLDL)	[83]
voltage-gated Ca^2+^ channels	Yes	?	Yes (Ca^2+)^	[84]
ClC-3 channel	Yes	?	?	[85]
K_ca_2.3 channel	Yes	?	?	[86]
GABA receptor B	Yes	?	?	[87]
VGLUT1	Yes	Yes	?	[88]
AMPAR	Yes	Yes	?	[91,93]
ADAM 9	Yes	?	?	[95]
ADAM 15	Yes	?	?	[95]
ADAM 19	Yes	?	?	[95]
MT1-MMP	?	Yes	?	[53]
Enterovirus 71	?	Yes	?	[96]
Cytomegalovirus	Yes	?	?	[97]
Moloney murine leukemia virus	Yes	?	?	[98]

Casamento and Boucrot Table 1. Cargoes that may use FEME to enter cells.

Receptors, channels, and pathogens and viruses that bind to Endophilin enter cells in an Endophilin-dependent manner and/or are internalized in a ligand-dependent manner are all putative FEME cargoes. oxLDL: oxidized low-density lipoprotein; voltage-gated Ca^2+^ channels (L, N and P/Q types); ClC-3: Chloride ClC-3 channel; K_ca_2.3 channel: small-conductance calcium activated K_ca_2.3 channel; GABA receptor B: gamma-aminobutiric acid GABA receptor B (a ligand-gated Chloride channel); VGLUT1: Vesicular glutamate transporter 1; AMPAR: α-amino-3-hydroxy-5-methyl-4-isoxazolepropionic acid receptor; ADAM9, 15 and 19: disintegrin and metalloproteinase domain-containing proteins 9, 15 and 19; MT1-MMP: membrane-type matrix metalloproteinase 1.

Vesicular glutamate transporter 1 (VGLUT1) is known to bind directly to the SH3 domain of Endophilin through a PRM in its C-terminus that is absent from VGLUT2 or 3 [88,89] (Table 1). This is reminiscent of the binding of Endophilin to a PRM within a cytoplasmic loop of β1- but not β2- or β3-adrenergic receptors [35]. The binding of VGLUT1 to Endophilin recruits the transporter to a fast endocytic pathway and synaptic vesicle recycling, thereby regulating neurotransmitter release and short-term plasticity [88,90]. Endophilin also binds to the α-amino-3-hydroxy-5-methyl-4-isoxazolepropionic acid (AMPA) receptor, either directly to the GluA1, but not GluA2 subunit, or through the adaptor Arc/Arg3.1 [91–93] (Table 1). The absence of Arc or Endophilin reduces AMPAR uptake and increases cell surface levels of the receptor, thereby affecting post-synaptic neuron plasticity and long-term depression. Although UFE — which is Endophilin-dependent as well — could be quickly recycling VGLUT1, this pathway has not been identified on the post post-synaptic side, where AMPA receptors are located. Thus, this opens the possibility that UFE and FEME function on the pre- and post-synaptic sides, respectively. However, before synapse formation, a Clathrin-independent, Endophilin-dependent endocytic pathway reminiscent to FEME controls rapid vesicle production at growth cones [94]. This is consistent with the recent finding that axon guidance receptors PlexinA1 and ROBO1 are FEME cargoes [28].

Endophilin binds to the disintegrin and metalloproteinase domain-containing proteins ADAM 9, 15 and 19, but not ADAM10, 12 or TACE [95] (Table 1). In addition, the Endophilin A2 SH3 domain interacts with a PRM within FAK, which supports the phosphorylation of Endophilin by Scr at Tyr315 [53]. The phosphorylation inhibits the binding of Endophilin to Dynamin as well the uptake of the membrane-type matrix metalloproteinase 1 (MT1-MMP) [53]. Elevated levels of MT1-MMP at the cell surface then increase extracellular matrix degradation and migration. Thus, Endophilin might sort metalloproteinases into FEME carriers. The prominence of FEME at the leading edges of migrating cells is coherent with that hypothesis.

In addition to endogenous channels and receptors, Endophilin is also known to bind to viral proteins or mediate the entry of viruses. Enterovirus 71 entry into cells requires Dynamin and Endophilin A2 (Table 1), in a pathway the authors called Endophilin-Mediated Endocytosis (EME), which may very well be the same as FEME [96]. Endophilin A2 also interacts with mouse cytomegalovirus egress protein pM50 [97] and Moloney murine leukemia virus Gag [98] (Table 1).

Finally, the recent identification of Bin1 as a FEME component opens up possibilities. Its SH3 domain binds to different PRMs to those of Endophilin and may sort different sets of cargoes into FEME carriers. So far, only integrin α3 has been reported to bind to Bin1 [99], but it is not clear whether the interaction is linked to endocytosis. Because of the poor solubility of transmembrane proteins, they are underrepresented in immuno-precipitation and pull-down experiments performed with classical detergents (e.g. Triton X-100) and many Endophilin or Bin1 interactors may have escaped detection. Targeted efforts are required to identify new FEME cargoes.

## Pending questions and future milestones

There are many questions that need to be answered to increase our fundamental understanding of FEME. In this section, we stand on recent literature to ask five questions, answers to which would constitute important milestones in our understanding of the molecular mechanism and physiological functions of FEME.

### How are initiation, cargo sorting, curvature and scission co-ordinated?

Several mechanistic details about FEME carrier formation are still missing. First, it is not clear when membrane curvature starts. During initiation, Endophilin is assembled into transient and dynamic patches, but there is no detectable curvature at this stage by immuno-electron microscopy [16]. At this stage, F-BAR and N-BAR domain proteins FBP17 and CIP4, Bin1, SH3BP1 and RICH1 are also present at initiation patches (albeit with different timings of arrival) [18], but they do not appear sufficient to induce significant membrane curvature. It is only after receptor stimulation that membrane bending is observed and tubules form [16]. It could be that cargo sorting brings the local copy number of Endophilin over a threshold triggering membrane curvature. Interestingly, if membrane curvature is induced independently of Endophilin, as it is the case during Shiga and cholera toxin entry where the toxins induce membrane bending upon clustering on the extracellular leaflet of the plasma membrane [38], then the need for receptor activation is bypassed [17]. Endophilin then functions during membrane scission, together with Dynamin and actin [17]. Overexpression of Endophilin (and of other N-BAR domain proteins) has long been known to be sufficient to form membrane tubules [100,101]. However, because Endophilin clustering into the initiation spots is SH3- and not N-BAR-domain dependent [18], it is not clear when the N-BAR domains become engaged with the membrane. It is important to remember that ectopically expressed N-BAR domains heterodimerize with endogenous full-length Endophilin molecules [16]. Therefore, papers claiming that the N-BAR domain is sufficient on its own for membrane targeting and bending *in vivo* must be interpreted with caution. Precise measurements of the number of Endophilin and Bin1 molecules at each stage of FEME carrier formation would likely provide further information on the process.

The mechanism of FEME carrier detachment is better understood but the timing, stoichiometries and individual contributions from (i) the friction imposed by the N-BAR domains of Endophilin and Bin1, (ii) the membrane tension enforced by actin and Dynein, and (iii) the membrane scission activity of Dynamin need to be worked out. For example, the role of Dynamin is not entirely clear. It is obviously required as any genetic or chemical inhibition methods tested so far blocked FEME carrier budding [16,17]. But Dynamin is both recruited onto initiation platforms in resting cells and on forming FEME carrier upon stimulation [18]. It is possible that it has a dual function as it does during CME [102]. Moreover, Endophilin blocks Dynamin-mediated membrane fission at high local concentration [42], suggesting a mechanism to exclude Dynamin from forming tubules and restricts its localization to their extremities. The timing of recruitment and localization of Bin1 and Dynein on FEME carriers (are they localized at the opposite end of the tubules rather than at the plasma membrane?) will be important to figure out. Finally, the mechanism, orientation and timing of actin polymerization around budding FEME carriers will require detailed and careful work, but will complete the picture.

### How are inhibitory phosphorylations removed?

FEME is held off by several phosphorylations imposed on cargoes, Dynein and Dynamin by Cdk5, GSK3β, and on Endophilin by Src, ROCK, DYRK1A and LRRK2 [16,53–55]. The promptness of FEME activation upon stimulation suggests that phosphatases quickly erase inhibitory phosphorylations. However, it is not obvious which phosphatase(s) could be acting downstream of the FEME cargoes known to date, which are as diverse as Gα_s_- and Gα_i_-coupled GPCRs, RTKs, cytokine or cell guidance receptors. In addition, kinases other than Cdk5 are likely priming GSK3β phosphorylation during FEME. In synapses, several endocytic proteins, including Endophilin, Dynamin and Amphiphysin, are targets of Cdk5 and GSK3β [49,103]. Their acute dephosphorylation upon axon depolarization is mediated by the Calcineurin phosphatase, which is activated by the sudden Ca^2+^ rise. Swift removal of the inhibitory phosphorylations then quickly activates compensatory endocytosis [104,105]. In non-neuronal cells, it is not known whether Ca^2+^ controls FEME. But it does mediate NCE of EGFR through Reticulon-3-dependent ER-PM contact sites [106]. As FEME is very polarized (i.e. it occurs on discrete location on the plasma membrane), it is unlikely that it is activated by a global and diffuse Ca^2+^ rise. Local Ca^2+^ increases happen in confined environments, such as ER-PM or Mitochondria-PM contact sites. But so far, none of these were observed around FEME carriers, nor were their requirements tested.

Another hypothesis is that generic phosphatases constitutively remove the inhibitory phosphorylations and that the regulation is at the level of the kinases — as long as they are active and keep phosphorylating the targets, the pathway is blocked. As soon as one, or several, of the kinases are inhibited and stop adding phosphate groups, the balance shifts under the remaining action of the phosphatases. GSK3β is an obvious candidate as it is inactivated upon the phosphorylation of Ser9 in its disordered N-terminus. The phosphorylation induces an auto-inhibition of GSK3β, as it mimics a priming phosphorylated Serine, and occupies the docking site, thereby blocking interactions with substrates [75]. Many kinases that are activated by growth factor receptor signaling, including AKT and ERK, phosphorylate Ser9-GSK3β [75,107]. In that model, receptor stimulation would trigger the activation of kinase(s) that inactivate GSK3β, thus relieving the negative pressure on FEME. Constitutive phosphatases would then quickly remove key inhibitory phosphorylations and trigger FEME. This model is supported by the lower level of regulation on phosphatases and the constitutive activity of many of them. Deciphering between these two hypotheses should inform us on a cardinal feature of FEME.

### What is the fate of the FEME cargoes?

The current understanding is that, whatever the endocytic pathways used to depart from the plasma membrane, all cargoes end up in early endosomes (defined as Rab5- and EEA1-positive endosomes). Some cargoes transit through APPL1 and 2-positive compartments before reaching early endosomes [108]. From there, cargoes are either sorted into late endosomes and lysosomes or recycled back to the plasma membrane, via few different paths [109]. The two options lead to radically different fates (degradation or recovery, respectively). The generic destination of cargoes that enter cells through FEME is not known yet. Shiga and cholera toxins escape the degrading pathway to reach the Golgi complex and endoplasmic reticulum [110]. Endogenous β1 adrenergic receptor is found in LAMP-1-positive late endosomes and lysosomes 30 min post-stimulation and its cellular uptake is almost entirely dependent on FEME [16,18]. Endogenous EGFR receptor enters cells through FEME carriers and ends up into lysosomes upon stimulation with high (>50 ng/ml) EGF levels. But inhibition of FEME does not affect EGFR total uptake or its degradation, consistent with the existence of alternative endocytic routes (EGFR-NCE and macropinocytosis). The fate of other FEME cargoes has not been measured precisely to date. The exercise is complicated by the loss of Endophilin staining upon FEME carrier fusion with endosomes, owing to the transformation of Pi(3,4)P_2_ into Pi(3)P by PTEN, and INPP4A and B phosphatases [45,111]. Following cargoes that enter cells through several parallel endocytic routes (such as EGFR) carries the risk of accounting for molecules that were endocytosed by other pathways than FEME.

But should all FEME cargoes end up being sent for degradation, one could ask about the need for a fast endocytic pathway for the very first step, as early endosome to lysosome maturation lasts at least 20 min [112,113]. An exciting hypothesis is whether FEME could deliver cargoes directly to late endosomes for expedited degradation, bypassing early endosomes. Indeed, in INPP4A and B depleted cells, Endophilin staining is found on LAMP1-positive late, not EEA1-positive early endosomes [16] and Endophilin binds to ESCRT-I components ALIX and Tsg101 [18,114]. Endophilin also recruits the ubiquitin ligase Cbl (through CIN85) to EGFR and HGFR [36,37], thereby potentially linking mono-ubiquitination (the ESCRT sorting signal) and FEME carrier formation. Alternatively, as Endophilin (both A and B subtypes) has been involved in autophagosome formation and protein degradation [115–117], it is tempting to speculate that FEME carriers mature into atypical autophagosomes for accelerated delivery into lysosomes. However, molecular details and direct evidence to support either hypothesis are lacking so far. Whatever the mechanism, future work uncovering the fate of FEME cargoes will set a milestone in our understanding of the pathway.

### Is FEME involved in neurodegenerative diseases?

There is evidence that the two main FEME components Endophilin and Bin1 are involved in neurodegeneration, in particular Alzheimer's and Parkinson's diseases. Endophilin A1 levels are elevated in Alzheimer's patients and transgenic mouse models [118]. Consistently, EndoA1 overexpression augments cerebral amyloid-β and increases neuronal death [119] and its depletion prevents synaptic disfunction induced by oligomeric amyloid-β [120]. In addition, LRRK2 is one of the small numbers of proteins shown to cause autosomal Parkinson's disease, mostly upon Gly2019Ser mutation that enhances its kinase activity [121]. LRRK2 phosphophorylates Endophilin at Thr73 and Ser75, which are located in the N-BAR domain, and thus decreases membrane binding and affects synaptic vesicle endocytosis [55,56]. Thus, one could speculate that impairment of UFE and/or FEME, which are both Endophilin-dependent, play a role in Parkinson's disease. However, considering the implication of Endophilin proteins in autophagosome formation and the strong link between autophagy and neurodegeneration, additional defects might induce the diseases.

The recent identification of Bin1 as a FEME component [28] broadens the possibilities for a link between FEME and neurodegeneration. Bin1 is strongly linked to Alzheimer's disease, but through Tau and amyloid-β pathology [122,123]. As for Endophilin, Bin1 levels are increased in Alzheimer's patients and its depletion suppressed Tau-mediated neurotoxicity [124]. However, lower Bin1 levels promote propagation of Tau pathology by decreasing aggregate endocytosis and lysosomal degradation [125]. In addition, Bin1 depletion increased BACE1 (because of decreased lysosomal targeting and degradation), resulting in elevated amyloid-β production [126]. Although a link between FEME and neurodegenerative diseases is yet to be established, there are several indications that the pathway may be involved. Its study holds promises for deepening our understanding of the pathologies and for potential new therapeutic avenues.

### Can FEME be targeted to potentiate anti-cancer treatments?

Endophilin is involved in several cancers. Its role appears complex, however, as both increased and decreased levels were measured in tumors. Moreover, Endophilin A2 (also known as extra eleven nineteen, EEN) is a fusion partner of the mixed-lineage leukemia protein (MLL) in human acute leukemia [127]. Endophilin loss of expression in bladder cancer correlates with tumor progression [128]. Consistent with a role for FEME in EGFR signaling [16], the stable silencing of Endophilin increases phosphorylation of AKT, GSK3β, SFK and STAT3 after EGF stimulation, mimicking the signaling pattern in urothelial carcinoma [128]. Interestingly, Endophilin stimulates cell migration by binding to RacGEF TIAM1 and potentiates colon cancer metastasis [129]. Conversely, Endophilin depletion blunts cell migration [16,27], and may limit tumor spreading. In addition, FEME priming proteins SHIP2, FBP17, CIP4, Lamellipodin and SH3BP1 all promote tumor metastasis in several types of cancer, including lung and breast cancer [130–135].

In other cancers, such as Human epidermal growth factor receptor 2 (HER2)-positive tumors, Endophilin levels are elevated [136]. Endophilin knock-down reduced HER2 uptake and elevated the levels of HER2 at the plasma membrane. This decreased AKT and ERK downstream signaling as well as migration and metastasis of HER2+ cancer cells. Interestingly, elevated HER2 levels at the cell surface upon Endophilin depletion made cells more sensitive to trastuzumab and trastuzumab-emtansine (T-DM1) antibody therapies [136]. As several receptors, including EGFR, accumulate at the cell surface upon FEME inhibition [16], the possibility that blocking the pathway may potentiate anti-cancer therapies is exciting. Recently, stopping endocytosis using Dynamin but not Clathrin inhibitors was shown to improve antibody-dependent cellular cytotoxicity (ADCC) to the anti-EGFR, anti-Her2 and anti-PD-L1 monoclonal antibodies (cetuximab, trastuzumab and avelumab, respectively) [137]. ADCC is mediated by the recognition of opsonized targets by FcgRs and natural killer (NK) cells, which kill their target using perforin and granzymes [138]. For ADCC to occur, the antigen-mAb complex must remain on the cell surface long enough to engage the interaction of the mAb Fc region and cytotoxic cells [139]. Interestingly, FEME was identified as the main endocytic pathway inhibited alongside CME in Dynamin-inhibited cells [137]. One of the Dynamin drugs, Prochlorperazine (PCZ) [80], has been used clinically in humans for many years and the efficacy and safety of the combination of PCZ with monoclonal antibody therapy was demonstrated in Phase 1B clinical trials [137]. PCZ blocks FEME [16] and was used to control epilepsy [140], chronic kidney disease [141], Candida and viral infections [142–144], opening the possibility that dampening FEME may be beneficial in treating cancers as well as other diseases.

## Conclusion and perspectives

FEME may be a major Clathrin-independent, Dynamin-dependent endocytic pathway and may emerge as an important therapeutic target. FEME was not characterized until few years ago, perhaps because it is not constitutively active and because it is only observed under physiological conditions (i.e. at 37°C and full serum media). Thus, the molecular mechanism is still being worked out and many aspects — some of which were discussed in this review — remain to be understood. Breakthrough in its understanding would arise from the discovery of ubiquitously expressed cargo receptor(s) that can easily be assayed, as transferrin receptor is for CME. Considerable advances may also come from the identification of specific cytosolic markers, if such proteins exist. Endophilin A1 to A3 being simple proteins binding to membranes via their N-BAR domains and to PRM-containing proteins through their SH3 domains, they are unlikely to be only functioning in FEME. Consistently, they have been involved in other cellular processes such as CME, some type of autophagy [115–117], as well as exocytosis of neurosecretory vesicles [146–148]. There are also probably important functional differences between the A1, A2 and A3 subtypes, as suggested by the recent finding that Endophilin A3, but not A2, controls the Clathrin-independent and Dynamin-independent uptake of CD166, induced upon extracellular clustering by Galectin-8 [145]. Finally, further investigation of the physiological functions supported by FEME should inform on new strategies to treat some of the corresponding diseases. The non-constitutive and regulated nature of the pathway is likely to be a precious asset to activate or inhibit on demand certain specific cellular processes, for the benefit of novel medical treatments.

## Open Access

Open access for this article was enabled by the participation of University College London in an all-inclusive *Read & Publish* pilot with Portland Press and the Biochemical Society under a transformative agreement with JISC.

## Abbreviations

ADBE: activity-dependent bulk endocytosis
ADCC: antibody-dependent cellular cytotoxicity
AMPA: α-amino-3-hydroxy-5-methyl-4-isoxazolepropionic acid
AR: adrenergic receptor
CIE: clathrin-independent endocytosis
CLIC: clathrin-independent carriers
CME: clathrin-mediated endocytosis
EGFR: epidermal growth factor receptor
EGFR-NCE: EGFR non-clathrin endocytosis
FDS: friction-driven scission
FEME: Fast Endophilin-Mediated Endocytosis
GABA: gamma-aminobutiric acid
GAPs: GTPase-activating proteins
HER2: human epidermal growth factor receptor 2
MEND: massive endocytosis
MT1-MMP: membrane-type matrix metalloproteinase 1
PCZ: prochlorperazine
RTK: receptor tyrosine kinase
SH3: SRC Homology 3
UFE: ultrafast endocytosis
VGLUT1: vesicular glutamate transporter 1
